# The use of mesoscale eddies by juvenile loggerhead sea turtles (*Caretta caretta*) in the southwestern Atlantic

**DOI:** 10.1371/journal.pone.0172839

**Published:** 2017-03-01

**Authors:** Peter Gaube, Caren Barceló, Dennis J. McGillicuddy, Andrés Domingo, Philip Miller, Bruno Giffoni, Neca Marcovaldi, Yonat Swimmer

**Affiliations:** 1 Department of Applied Ocean Physics and Engineering, Woods Hole Oceanographic Institution, Woods Hole, Massachusetts, United States of America; 2 College of Earth, Ocean and Atmospheric Sciences, Oregon State University, Corvallis, Oregon, United States of America; 3 Dirección Nacional de Recursos Acuáticos, Montevideo, Uruguay; 4 Centro de Investigación y Conservación Marina (CICMAR), El Pinar, Canelones, Uruguay; 5 Projecto TAMAR, Fundação Pró Tamar / ICMBio, Salvador, Bahia, Brazil; 6 NOAA Fisheries, Long Beach, California, United States of America; University of California Santa Cruz, UNITED STATES

## Abstract

Marine animals, such as turtles, seabirds and pelagic fishes, are observed to travel and congregate around eddies in the open ocean. Mesoscale eddies, large swirling ocean vortices with radius scales of approximately 50–100 *km*, provide environmental variability that can structure these populations. In this study, we investigate the use of mesoscale eddies by 24 individual juvenile loggerhead sea turtles (*Caretta caretta)* in the Brazil-Malvinas Confluence region. The influence of eddies on turtles is assessed by collocating the turtle trajectories to the tracks of mesoscale eddies identified in maps of sea level anomaly. Juvenile loggerhead sea turtles are significantly more likely to be located in the interiors of anticyclones in this region. The distribution of surface drifters in eddy interiors reveals no significant association with the interiors of cyclones or anticyclones, suggesting higher prevalence of turtles in anticyclones is a result of their behavior. In the southern portion of the Brazil-Malvinas Confluence region, turtle swimming speed is significantly slower in the interiors of anticyclones, when compared to the periphery, suggesting that these turtles are possibly feeding on prey items associated with anomalously low near-surface chlorophyll concentrations observed in those features.

## Introduction

Mesoscale eddies have been identified as “hot spots” of biological activity, spanning trophic levels from primary producers [1–4] to zooplankton and small fish [5], up to large pelagic fish [6]. Recent advances in satellite oceanography have allowed the automated identification and tracking of mesoscale ocean eddies globally [7]. These advances in our ability to observe and track eddies has enabled us to analyze their impact on marine biota, revealing rich regional variability in how eddies influence near-surface chlorophyll (CHL) distributions [8, 9] and structure populations of animals (e.g., [10]).

Furthering our understanding of how ocean current influence the distribution of marine animals, however, remains a major challenge in the field of pelagic ecology. Significant progress can now be gained via the development of analysis techniques that aim to elucidate how animals use oceanographic features, such as eddies and fronts, which has only recently been possible with the advent of mesoscale feature tracking techniques described above. For example, in the Atlantic Ocean, Mansfield et al. [11] affixed small solar powered satellite transmitters to neonate logger head turtles (carapace length 11–18 cm) observing migrations that spanned thousands of kilometers with individuals spending on average nearly 70% of their time swimming in mesoscale eddies shed from the Gulf Stream. In the northwestern Pacific, along the Kuroshio Extension, Polovina et al. [12] observed juvenile loggerhead turtles interacting with the peripheries of eddies, at times swimming against the prevailing current. The authors suggested that the foraging advantage associated with eddy currents exceed the energetic demand associated with swimming against the current. A series of studies of the post nesting migration of loggerhead sea turtles revealed that in the Gulf of Mexico, North Pacific, and North Atlantic, turtles displayed a preference for the periphery of open-ocean eddies, suggesting that these turtles were foraging in those areas [13, 14]. The aforementioned studies were conducted in a turtle-centric frame of reference, collocating satellite observations to the turtle trajectories. In a region of high abundance between Taiwan and China (referred to as a turtle “hotspot”), Kobayashi et al. [10] analyzed the trajectories of 34 non-reproductive loggerhead sea turtles by collocating their locations to both the centers and peripheries of mesoscale eddies. Their study revealed that the turtles avoided the peripheries of cyclonic eddies, in contrast to what was observed in other regions.

The studies described above conclude that turtles use different parts of eddies depending on their geographic location or life stage. In this study, we investigate the use of eddies by juvenile loggerhead sea turtles in the Brazil-Malvinas Confluence (BMC) region by collocating the daily positions of 24 individual turtles with eddies identified and tracked in maps of sea level anomaly (SLA). Our investigation aims to: 1) characterize the distributions of eddy polarity, amplitude, radius, and lifetime in the BMC region; 2) investigate the influence of eddies on CHL and sea surface temperature (SST) in this region; and 3) compare the distribution of turtles relative to passive Lagrangian drifters in regard to eddies, which has been shown to allow for the determination of the relative roles of active changes in turtle swimming behavior to passive advection of turtles by the ambient ocean surface currents [15]. Specifically, we seek to understand if loggerhead turtles in this region are modifying their behavior to interact with and remain in eddies, or whether their distribution is the result of the passive advection.

## Methods

### Sea level anomaly, geostrophic velocities and rddy identification

This investigation of the use of mesoscale eddies by juvenile loggerhead sea turtles was based on eddies with lifetimes of 12 weeks (84 days) and longer that have been identified and tracked based on their signatures in SLA [7]. The altimeter-tracked eddy dataset used in this analysis is available online at *http://cioss.coas.oregonstate.edu/eddies*. The SLA fields were obtained from Collecte Localis Satellites (CLS/AVISO, *http://www.aviso.altimetry.fr*) at 7-day intervals on a 1/4° latitude by 1/4° longitude grid. Prior to the identification and tracking of mesoscale eddies, the SLA fields were high-pass filtered to remove the effects of seasonal heating and cooling [7].

In order to infer swimming behavior of the turtles (see 2.2 below), geostrophic current velocities were estimated by centered finite differencing of the SLA fields:

$$u_{g}=-\frac{g}{f}\frac{\partial \mathrm{SLA}}{\partial y}$$ (1)

$$v_{g}=\frac{g}{f}\frac{\partial \mathrm{SLA}}{\partial x}$$ (2)

where *g* is the gravational constant and *f* = 2Ω sin Φ is the Coriolis parameter for latitude Φ and Earth rotation rate Ω and *x* and *y* are the zonal and meridional coordinate, respectively.

As described in detail in Appendix B of [7], mesoscale eddies were identified and tracked based on closed contours of SLA. The eddy amplitude at each weekly time step along its trajectory was defined as the difference between the SLA at the eddy centroid and around the outermost closed contour of SLA. The characteristic rotational speed of each eddy (U) was defined as the average geostrophic speed along the SLA contour around which U is maximum, and was computed at each point along the eddy trajectory. The horizontal speed-based radius scale of the eddy (L_s_) was defined to be the radius of a circle with area equal to that enclosed by this SLA contour. The eddy propagation speed was estimated at each point along the eddy trajectory from centered differences of the *x* and *y* coordinates of successive eddy SLA centroid locations.

**Fig 1:**
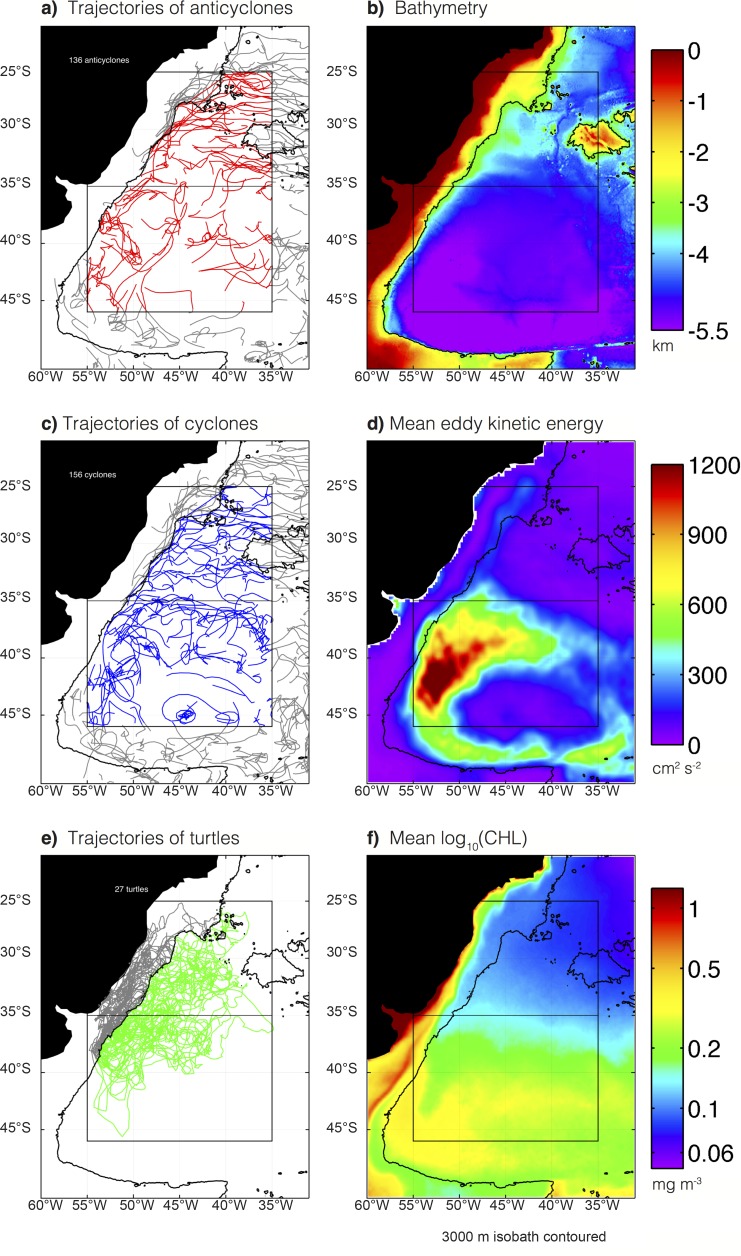
Maps of the study region. (A) Trajectories of anticyclonic mesoscale eddies during the 5-year study period. Red trajectories are of eddies investigated in this study, grey trajectories are of eddies surrounding the region of interest, but not used in the analysis. (B) Bathymetric map of the study region. (C) Same as panel a, but for cyclonic eddies. (D) Mean eddy kinetic energy from satellite *SLA* observations. (E) Tracks of the 24 turtles. Grey trajectories indicate when the turtles were observed to be shallower than 3,000m, and were excluded from the analysis here. (F) Mean of the log_10_ transformed *CHL*. The 3,000 m isobath is contoured in all panels.

In order to avoid the complexities of coastal habitats, we considered areas with water depth in excess 3,000 m, as defined by the National Oceanic and Atmospheric Administration (NOAA), National Geophysical Data Center, 2-minute Gridded Global Relief Data (ETOPO2v2, *https://www.ngdc.noaa.gov/mgg/global/etopo2.html*) (Fig 1B).

### Tracking turtles

Scientific observers of PNOFA-DINARA (the Uruguayan National Program of Scientific Observers Onboard the Tuna Fleet) [16, 17] and Projeto TAMAR-ICMBio (the national Brazilian sea turtle conservation program) [18] deployed satellite transmitters on loggerhead sea turtles incidentally captured in Brazilian and Uruguayan pelagic long-line fisheries operating in the southwestern Atlantic Ocean between July of 2006 and November of 2009. The turtle trajectories used in this study span a 5-year period between July 2006 and December 2011(Fig 1E) and are available in S1 File.

As described in [16] satellite transmitters were attached to the turtles on the second central carapacial scute using quick-drying two-part epoxies, *PoxipolTM* (Uruguay) and *DurepoxiTM* (Brazil), and allowed to dry for 30 minutes to one hour on deck before release. A total of 24 tracked loggerheads with mean curved carapace length of 61.8 ± 6.9 cm (range: 49 to 83 cm, see Table 1 of [15]) that transmitted more than 30 days are included in this study. ARGOS-linked *Telonics* (Mesa, AZ, USA) platform transmitter terminals (*PTTs*), models ST-18 and ST-20, were attached to 4 and 5 turtles, respectively, on Brazilian vessels. *ARGOS-linked Wildlife Computers* (Redmond, WA, USA) PTTs, models *SPLASH* and *SPOT 5*, were attached to 5 and 10 turtles, respectively, on Uruguayan vessels. See [16] for further turtle tracking details.

The first ten days (3% of total points) of tracking data were excluded from each turtle in order to avoid including immediate post-release behavior that may have been affected by the capture event. We only considered individual turtle locations with ARGOS estimated errors of <1500 m of the tag’s actual position (location classes 1–3, ARGOS 2008). Tracking data were downloaded and filtered using the Satellite Tracking and Analyst Tool [19]. We used single daily locations in order to reduce spatial autocorrelation [20–22]. Only daily turtle locations that had observed turtle propagation speed of < 5 km h^-1^ from one day to the next are retained [20, 21]. The speed-based thresholding results in the removal of 1% of the total turtle locations.

Turtle trajectories represent the summation of both turtle swimming behavior and ocean surface currents [23]. The total vector turtle velocity (*u_t_, v_t_*) was defined as the velocity components estimated from the distance an individual turtle travels between successive daily locations. Similar to Putman and Mansfield (24), the turtle swimming speed V_s_ was defined as

$$V_{s}=\sqrt{{\left(u_{t}-u_{g}\right)}^{2}+{\left(v_{t}-v_{g}\right)}^{2}}.$$ (3)

This formulation assumes that the fluid motions are purely geostrophic. Of course there are departures from geostrophy, but in the open ocean ageostrophic flows tend to be much weaker than the geostrophic component. In the near-surface region where the turtles reside, the largest source of ageostrophic flow is wind-driven motion. Across the BMC region, the long-term average wind-driven surface currents estimated from scatterometer data, as described in [8], range from 8 cm s^-1^ to 16 cm s^-1^ (not shown) and the wind direction is highly variable (see Fig 12 in [8]). Typical geostrophic flows are several times larger and more persistent in time, thus the wind-driven component can be thought of as high-frequency noise on the signal of interest. Moreover, the average magnitude of the turtle movement vectors is ~70 cm s^-1^, suggesting that to first order the approximation in Eq (3) is valid: V_s_ is a function of turtle behavior, and not passive advection by wind-driven currents.

### Drifters

The surface drifter data set was acquired from NOAA’s Atlantic Oceanographic and Meteorological Laboratory (*ftp://ftp.aoml.noaa.gov/pub/phod/buoydata/hourly_product*). A total of 282 drifters were extracted within the study region during the 5-year study period. Drifters are equipped with an approximately 5m long holey-sock drogue that is centered at 15m which allows the drifter to follow near-surface currents with minimal wind slip. Zonal and meridional root-mean-square errors in the satellite location fix are 630 m and 270 m, respectively (see overview of the global drifter program by [25]). Poor *ARGOS* locations were removed from the dataset and the trajectory of each drifter was created by optimal interpolation at uniform *6 hour* intervals following AOML procedures [26]. The four-times-a-day drifter locations were then averaged to once-per-day to match the temporal resolution of the turtle tracks.

### Near-surface chlorophyll concentration

This study uses the merged SeaWiFS, MODIS-Aqua and MERIS ocean color measurements. Near-surface chlorophyll pigment concentrations (CHL) were estimated from ocean color measurements using the Garver-Siegel-Maritorena (GSM) semi-analytical ocean color algorithm [27–29] with data available at *ftp://ftp.oceancolor.ucsb.edu//pub/org/oceancolor/MEaSUREs/MergedSAM/*. The detailed description of the processing details of the CHL observations can be found in [30]. Chlorophyll anomaly fields (CHL') were defined as:

$${\mathrm{CHL}}^{\prime }=\mathrm{CHL}-\langle \mathrm{CHL}\rangle$$ (4)

where ⟨CHL⟩ denotes 6° × 6° spatially smoothed fields that are removed from the total fields to create the anomalies.

Ambient CHL varies by more than an order of magnitude over the region of interest (Fig 1F), resulting in inhomogeneities of the CHL anomalies. To help mitigate the effects of geographical inhomogeneity in the anomaly fields, we normalized the anomalies at longitude *x* and latitude *y* by the long-term averaged background fields $\bar{\mathrm{CHL}\left(x,y\right)}$ at the same location,

$$\mathrm{CHL}"=\frac{\mathrm{CHL}\prime \left(x,y\right)}{\bar{\mathrm{CHL}\left(x,y\right)}}.$$ (5)

The normalized CHL anomalies are denoted by the double-primes and henceforth referred to as CHL''.

### Sea surface temperature observations

The sea surface temperature (SST) fields used here are the optimally interpolated SST analyses produced by the NOAA National Climatic Data Center. Microwave and infrared satellite observations are combined with *in situ* measurements of SST to obtain daily, global fields on a 1/4° latitude by 1/4° longitude grid [31]. The data is publically available at *ftp://eclipse.ncdc.noaa.gov/pub/OI-daily-v2/IEEE/*. To isolate variability predominantly at the oceanic mesoscale, the daily SST fields were spatially filtered. SST anomaly fields (SST') were computed as:

$${\mathrm{SST}}^{'}=\mathrm{SST}-\langle \mathrm{SST}\rangle$$ (6)

where ⟨SST⟩ denotes 6° × 6° spatially smoothed fields. Unlike CHL, normalization of the anomaly fields by the mean SST was not necessary because the background varies less (a factor of two for SST versus an order of magnitude for CHL). It is important to note that the analysis presented in section 3.2 was repeated on normalized SST anomalies and the results were qualitatively very similar (not shown).

### Collocating turtles, drifters and CHL to eddy interiors

Prior to collocating the locations of turtles and drifters with mesoscale eddies, the SLA, geostrophic velocities and eddy trajectories were interpolated from weekly to daily time steps using cubic-spline interpolation. Each daily turtle or drifter location was collocated to the closest eddy SLA extremum. To assess differences in the distribution of turtles in cyclonic and anticyclonic eddies, we constructed histograms of turtle location as a function of radial distance from the SLA extremum. These histograms were computed from the number of daily turtle locations per unit area of an annulus defined by the radial distance from the eddy centers.

**Fig 2:**
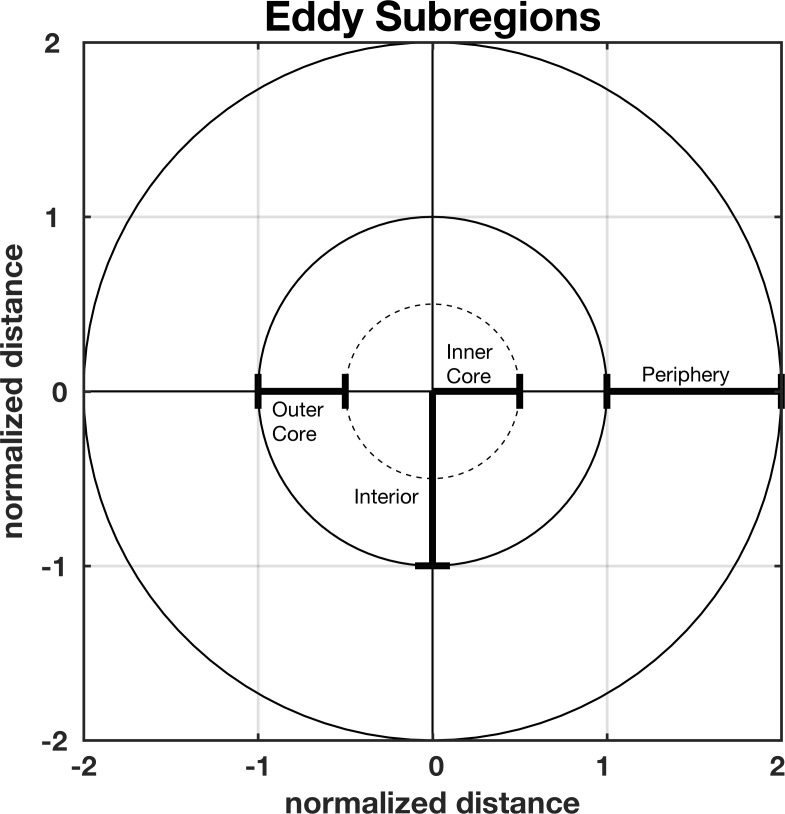
Eddy subregions. Schematic representation of the various eddy subregion defined in section 2.6. The *x* and *y* axis represent distance from the eddy center normalized by the eddy radius scale L_s_.

To investigate if turtles are more likely to be associated with the core, interior or periphery of cyclonic or anticyclonic eddies, we defined eddy subregions by the normalized distance r from the eddy SLA extremum (Fig 2). The eddy inner-core is defined as r ≤ L_s_/2. The outer core is defined as L_s_/2 < r ≤ L_s_ and the eddy interior is defined to include both the inner and outer core (r ≤ *L_s_*). The eddy periphery is defined as L_s_ < r ≤ 2L_s_, and the area outside of an eddy is defined as r > 2L_s_.

To assess the CHL and SST response to mesoscale eddies, composite medians were constructed following [30] by interpolating the satellite observations onto a common grid collocated to the eddy SLA extremum with distance scaled by the eddy radius L_s_. Each normalized grid location was then interpolated onto a high-resolution grid with zonal and meridional coordinates ranging from − 2L_s_ to 2L_s_. This normalization allowed composites to be constructed from hundreds to thousands of weekly eddy observations on a common grid defined by the horizontal size of each individual eddy. We used composite medians rather than composite averages because the latter are sometimes sensitive to occasional outliers in the anomalies of near-surface CHL.

## Results and discussion

### Eddies of the Brazil-Malvinas confluence region

Our study area is a region of active eddy generation [7]. The confluence of the Brazil and Malvinas currents spawns large amplitude eddies that propagate west and then south along the shelf break, with many eddies being advected eastward around the periphery of the Zapiola Gyre ([32–35]; Fig 1A and 1C). In total, there are 136 and 156 long-lived anticyclones and cyclones, respectively, during the 5-year study period.

The study region contains two distinct regimes of eddy kinetic energy (EKE) and CHL. The region to the north of 35S is fairly quiescent and relatively low in CHL (the mean time-averaged CHL is 0.11 mg m^-3^), whereas the region to the south is more energetic and contains more CHL (mean CHL of 0.25 mg m^-3^; Fig 1D and 1F). In light of the regional differences in the energetics of mesoscale eddies and the ambient CHL, eddies, turtle and drifter trajectories are analyzed separately for two subregions divided by 35°S.

**Fig 3:**
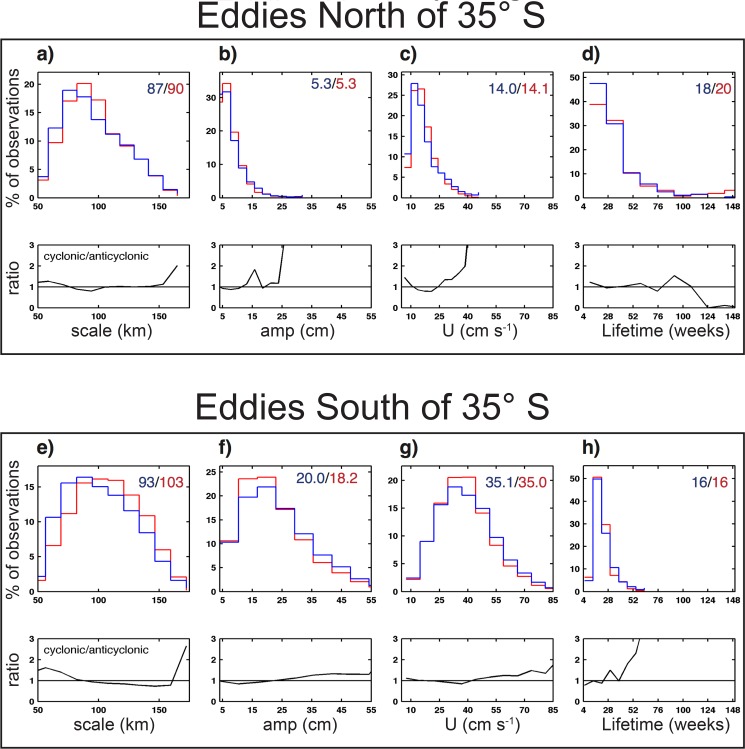
Histogram of eddy properties. Histograms of eddy properties are shown in the top row of each panel and the ratio of cyclonic to anticyclonic eddies is shown in the bottom row of each panel. (A) Speed-based eddy radial scale L_s_; (B) amplitude; (C) axial speed U and (D) lifetime of eddies within the study domain and north of 35°S. Cyclones and anticyclones shown as blue and red stepped curves, respectively. Panels (E) through (F) are the same as (A) through (D), except for eddies within the study domain and south of 35°S. Labels in the top right-hand corner of the top row of each panel are the median values of each panel shown in blue and red for cyclonic and anticyclonic eddies, respectively.

A total of 77 and 82 long-lived cyclones and anticyclones, respectively, were observed in the northern subregion during the 5-year study period (Fig 1A and 1C), resulting in a total of 1,260 and 1,362 weekly eddy observations. Eddies in the northern region were smaller in amplitude than those south of 35°S (Fig 3B and 3F). The median amplitude of northern eddies was 5.3 cm for both polarities (Fig 3B). The amplitude and radial scale of eddies in the northern region of our study domain were similar to the global average for the open ocean [7]. Eddies in this portion of the study region had median life times of 18 weeks and 20 weeks for cyclones and anticyclones, respectively (Fig 3D). An interesting distinction between anticyclonic and cyclonic eddies in this northern region is that the longest-lived eddies (lifetimes ≥ 124 weeks) were nearly all anticyclones (Fig 3D).

South of 35°S, a total of 79 and 48 long lived cyclones and anticyclones, respectively, were observed during the 5-year study period, resulting in a total of 1,297 and 796 weekly eddy observations. Eddies in this southern region were large in amplitude, with cyclones and anticyclones having median amplitudes of 20.0 cm and 18.2 cm, respectively (Fig 3F). More cyclones were observed to have amplitude ≥25 cm, which results in more cyclonic eddies with rotational velocities exceeding 45 cm s^-1^ (Fig 3G). Eddies in this southern region were shorter lived than to the north, with median lifetimes of 16 weeks for eddies of both polarities (Fig 3H).

### Eddy-induced perturbations of near-surface chlorophyll and sea surface temperature

**Fig 4:**
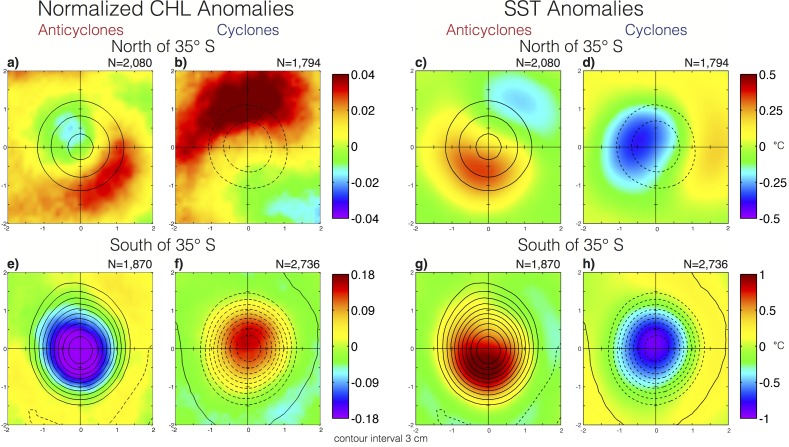
Eddy-centric composite of CHL and SST. Composite medians of CHL'' (left two columns) and SST anomalies (right two columns) for eddies in water depths in excess of 3,000 m. (A,C) Anticyclones and (B,D) cyclones north of 35°S. (E,G) Anticyclones and (F,H) cyclones south of 35°S. The *x* and *y* axes if each panel have been scaled by the horizontal speed-based eddy radial scale *L_s_*. The contours overlaid on each composite are the composite median of SLA at an interval of 3 cm, negative contours shown as dashed curves. Note different colorbar scaling used in the for observations north and south of 35°S.

To investigate the influence of eddies on CHL and SST, composites were constructed separately for anticyclones and cyclones in the northern and southern subregions. The SST' and CHL'' composites in the northern subregion were characterized primarily as having dipolar spatial structure (Fig 4A–4D), suggesting that these eddies primarily influence phytoplankton and the ambient SST fields by the advection of CHL and temperature gradients around their peripheries [8, 36]. It is important to note that differences between SST and CHL composites are observed, suggesting distinct mechanisms may dominate eddy-induced perturbations of SST and CHL in this region.

Composites of northern anticyclones contain a primary pole of positive CHL'' to the southeast of the composite eddy center with the largest positive CHL'' occurring along the periphery (Fig 4A). A secondary pole of negative CHL'' extends from the eddy core to the northwest. The SST anomalies of these anticyclones, however, are characterized by a primary pole of positive SST' that extends from the eddy core into the southwestern quadrant with the maximum of SST' occurring at a radial distance from the eddy center of 0.75L_s_ (Fig 4C). A secondary pole of negative SST' is located outside of the eddy periphery in the northeast quadrant. Northern cyclones contain a primary pole of elevated CHL to the north and a region of low CHL to southeast (Fig 4B). The composite SST' of these cyclones is characterized by a primary pole of negative SST' that extends from the eddy core into the northwest quadrant and a secondary pole of positive SST' to the east (Fig 4D). The dipole structure of the composite in the northern eddies suggests that eddy-induced perturbations of SST and CHL are primarily caused by advection of ambient gradients around the eddy periphery (“eddy stirring” [37]). It is important to note, however, that the anomalies are not zero at the eddy centers, as would be expected from the influence of eddy stirring alone, suggesting that other mechanisms (i.e. “eddy pumping” [34]) may be important in this region.

In the southern subregion, the observed geographical structure of eddy-induced CHL'' and SST' is best described as a monopole with negative CHL'' and positive SST' in the interiors of anticyclones (Fig 4E and 4G) and positive CHL'' with negative SST' within the interiors of cyclones (Fig 4F and 4H). The exact mechanism generating the observed monopoles of CHL'' and SST' in these eddies is ambiguous, as there are at least two processes that could be responsible for the observed patterns. In the case of CHL, Gaube et al. [37] showed that the observed CHL'' in these eddies is consistent with trapping of elevated or depressed CHL during formation of cyclones and anticyclones, respectively. Upwelling and downwelling occurring during the intensification of cyclones and anticyclones can also generate these same patterns in CHL'' and SST' via vertical displacement of the pycnocline and the associated effects on temperature and nutrient availability.

### Observations of juvenile loggerhead sea turtles and surface drifters in eddies

**Fig 5:**
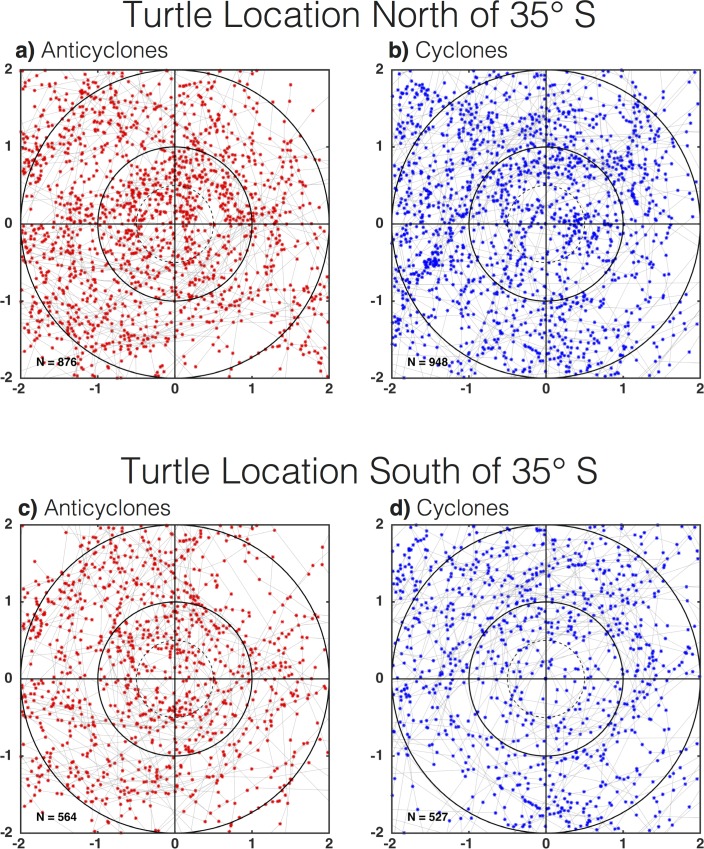
Eddy-centric turtle locations. Daily turtle locations collocated with the interiors of (A) anticyclonic and (B) cyclonic eddies north of 35°S, and (C) anticyclonic and (D) cyclonic eddies south of 35°S. The tracks of turtles in eddy-centric coordinates are shown as grey lines. The *x* and *y* axes if each panel have been scaled by the horizontal speed-based eddy radial scale L_s_ defined in section 2.1.

**Fig 6:**
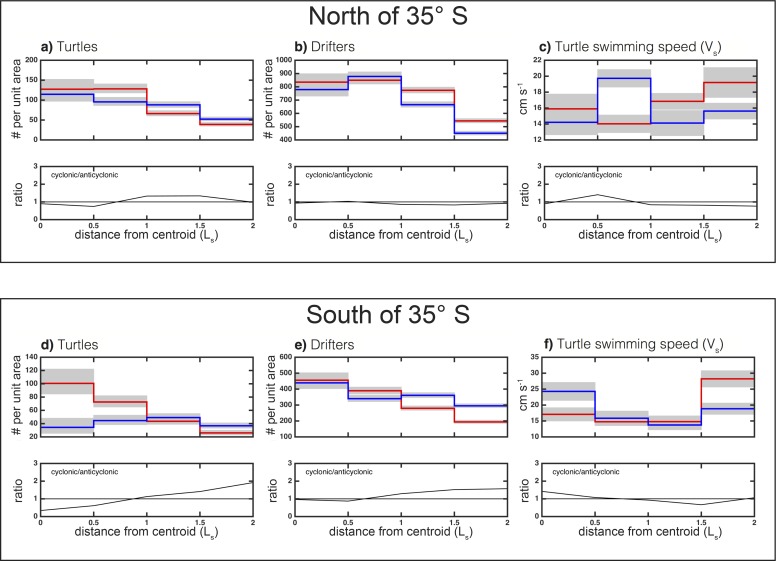
Normalized histograms of turtle, drifter and swimming speed. Histograms of the number of daily turtle locations (panels A and D) and drifter locations (panels B and E) per unit area of each radial annulus as a function of radial distance from the closest eddy centroid. Turtles and drifters associated with anticyclones and cyclones are shown as red and blue stepped curves, respectively. The *x* axes of each panel have been scaled by the horizontal speed-based eddy radial scale L_s_. The 95% confidence interval of each radial bin is shown by grey shading. Confidence intervals for the binomially distributed location counts were computed following [38]. The median turtle swimming speed (see section 2.2) as a function of radial distance from eddy centroids is shown in panels c and f. The 95% confidence interval of the mean is shown by grey shading in panels c and f.

In waters deeper than 3,000 m, turtles were observed to be within eddies (r ≤ 2L_s_) 65% of the time. In the northern subregion, a total of 876 and 948 daily turtle positions were observed within 2L_s_ of anticyclones and cyclones, respectively (Fig 5A and 5B). The binning of the daily positions observations as a function of distance from the eddy centers and normalization by the area of the annulus defined by each radial bin revealed that turtles were significantly more likely to be associated with the outer-cores of anticyclones than the outer-cores of cyclones (Fig 6A). When integrated over the eddy cores (distance *r* ≤ *L_s_*), the number of daily turtle locations per unit area was significantly larger in the interiors of anticyclones compared with cyclones (α = 0.1, not shown). Outside of the eddy core, turtles appeared to be associated more with the peripheries of cyclones than anticyclones. This raises the question, were turtles modifying their behavior to interact with and remain in these eddy subregions, or is this the result of the passive advection?

By repeating the above analysis on surface drifters, we were able to address the role of passive advection in structuring the observed distribution of turtles in different eddy subregions. It is important to note that drifters are constrained to the surface, whereas turtles are not, an important caveat noted by other studies [15]. Analysis of the pressure recording tags affixed to a subset of 5 turtles revealed that when the turtles were below the surface, they attained a maximum depth between 10 and 100 m 84% of the time (see [16] for a summary of dive depths for these turtles), suggesting that when diving, turtles often occupy the zone near where the drifters are drogued. Even in light of the differences in drifter configuration and turtle behavior, the comparison of the passive advection of drifters to the movement of turtles has yielded valuable insight into the active movement and foraging behavior of turtles (*e*.*g*., [39]).

Histograms of the number of drifters observed as a function of radial distance revealed no significant difference between the interiors of cyclones and anticyclones (Fig 6B). In the eddy peripheries, drifters were more likely to occupy anticyclones than cyclones, which was the opposite of what is observed for turtles (c.f. Fig 6A and 6B). These results suggest that juvenile loggerhead turtles in the BMC are not passively advected by ocean currents, which is not surprising as previous studies of turtles ranging from the neonatal stage and juveniles have shown that their trajectories differed substantially from those of passive drifters [24, 40, 41] and the paths of ambient ocean currents [21].

Another metric analyzed here to investigate if turtles modify their behavior to interact with or remain in particular regions of eddies is the median turtle swimming speed *V_s_* (Eq 3) when associated with eddies. Median *V_s_* ranged from ~ 14 cm s^-1^ to 30 cm s^-1^ (Fig 6C and 6F), which is slightly larger than the speeds reported for loggerhead turtle hatchlings [42, 43] and thus considered to be biologically feasible for juveniles of the same species. Turtles that were actively feeding, or were choosing to remain within a specific area, were expected to have a slower V_s_ than turtles that are seeking suitable foraging habitat. Although there were not significant differences in V_s_ in the inner cores of the two types of eddies, swimming speed was higher in the outer cores of cyclones (Fig 6C). This behavior may have contributed to the lower abundance of turtles in the outer cores of cyclones relative to that in anticyclones, given that the distributions of drifters in those same eddy subregions are equal. Swimming speed was also significantly elevated along the periphery of anticyclones when compared to cyclones (Fig 6C; note that the confidence intervals in the inner periphery are just barely distinct from each other), suggesting that turtles may try to avoid the peripheries of anticyclonic eddies. This hypothesis was supported by the relatively lower abundance of turtles in the peripheries of anticyclones, in contrast to the relatively higher abundance of drifters in those same eddy subregions.

In the southern subregion, a total of 564 and 527 individual daily turtle location estimates were observed within 2L_s_ of anticyclones and cyclones, respectively. The eddy-centric composites revealed an apparent association of turtles with the interiors of anticyclones and along the peripheries of cyclones (Fig 5C and 5D), which was confirmed in the radial histograms (Fig 6D; note that the differences outside the cores are only different in the outer peripheries). Drifters, on the other hand, were equally likely to be associated with the inner cores of anticyclones versus cyclones, and were only 5% more likely to be in the outer-core of anticyclones than cyclones (Fig 6E). Median V_s_ was significantly slower in the cores and inner peripheries of anticyclones, when compared to their outer peripheries (Fig 6F). This suggests that when turtles were in the cores and inner peripheries of anticyclones, they slowed their swimming, possibly in an attempt to remain in this eddy subregion. On the other hand, when turtles found themselves on the outer periphery of anticyclones, they elevated their swimming speed, possibly to move to a different eddy subregion. It is also interesting to note that median swimming speed was significantly elevated in the cores of cyclones (Fig 6F), which may reflect avoidance of those features.

A possible bias may have been introduced from the colocation of turtles to eddies with lifetimes greater than or equal to 12 weeks. A turtle could have been assigned to a particular long-lived eddy, when in fact it was closer to an ephemeral eddy, with lifetime shorter than 12 weeks. The analysis presented throughout this section was repeated using an eddy lifetime cutoff of 4 weeks, which did not yield any significant differences in the results. Therefore, we do not consider our results to be particularly sensitive to the collocation of turtles with long-lived eddies instead of ephemeral eddies with lifetimes shorter than 12 weeks.

### Possible mechanisms controlling the distribution of turtles in eddies

The most pronounced signal in the eddy-centric distribution of turtles is the apparent preference for the interiors of anticyclones in the southern region (Fig 6D), which were associated with low CHL'' and warm SST’ (Fig 4E and 4G). We suggest two possible hypotheses for the association of turtles with the cores of anticyclones south of 35°S: (1) Turtles were passively advected south 35°S in the interiors of anticyclones that propagate south until certain cues (perhaps temperature or elevated CHL) indicate the need for northward movement on the part of the turtle. (2) Turtles were actively modifying their behavior to interact with and remain in anticyclones south of 35°S possibly because of suitable foraging conditions associated with low CHL, decreased predation, elevated ambient temperatures which influence turtle growth rates, feeding behavior, movement speed and physiological and immune competence [44, 45] or other reasons not discernable from the data analyzed here.

If turtles were passively advected southward inside anticyclones, we expect the proportion of turtle locations in anticyclones to be higher during southward propagation across the 35°S boundary when compared to northward propagation across the boundary. Indeed, turtles are 11% more likely to be in anticyclones while moving southward versus northward. Thus, we conclude that turtles may be passively advected southward inside anticyclones, however, it is also possible that turtles modify their behavior to interact with the pelagic ecosystems trapped within these southern anticyclones.

In the southern subregion, V_S_ was significantly slower in the cores and inner peripheries of anticyclones, when compared to the outer peripheries (Fig 6F). This leads to the question, what are the physical and biological characteristics of anticyclones that could cause turtles to lower their swimming speed? The analysis of eddy-centric CHL and SST anomaly composites in section 3.2 revealed that south of 35°S the interiors of anticyclones and periphery of cyclones are characterized by low CHL and warm SST anomalies.

As turtles do not feed on CHL, they were likely preferentially seeking out some other environmental or biological property that is associated with low CHL and warm water observed in anticyclones in the southern BMC region. Numerous investigations of the stomach contents of deceased juvenile loggerhead sea turtles (size ranging from 5.2 to 30.0 cm strait carapace length) report a high concentration of gelatinous organisms contained therein (see review by Bjorndal et al. [46]). Parker at al. [47] found that *Janthina sp*. (a pelagic gastropod) was the most common prey item for oceanic stage loggerheads (size ranging from 13.5 to 74.0 cm curved carapace length) in the North Pacific followed by *Carinaria carinaria* (a gastropod) and a colonial hydroid *Velella velella*. In addition, Bowen et al. [48] found that in the Eastern Pacific, large aggregations of juvenile loggerhead sea turtles foraged on pelagic red crabs. In the North Atlantic, investigation of the stomach contents of 12 oceanic-stage loggerhead turtles concluded that the *V*. *velella* was the most abundant prey, both in terms of volume and frequency [49].

The particular properties of anticyclones in the southern regions that cue juvenile loggerhead turtles to remain within the eddy cores are not directly discernable from the data analyzed here. One possible mechanism is that enhanced foraging success in anticyclones, be it the result of elevated prey or the metabolic advantage associated with warmer water, results in turtles modifying their behavior to actively remaining in anticyclones.

### Comparison to previous investigations of the interaction turtles with eddies

In an investigation of non-reproductive loggerhead sea turtles in the East China Sea, Kobayashi et al. [10] computed the proximity-probability of turtles to the centers and peripheries of mesoscale eddies. Using this metric, they concluded that turtles avoided the peripheries of energetic cyclonic eddies. In the analysis presented here, we normalize the number of turtle colocations by the area of each radial band (see section 2.6), removing the bias resulting from the relatively large area encompassed by the eddy periphery compared to the core. Using this method, we find that turtles in the southern region are significantly more likely to associate with the interiors of anticyclonic eddies, contrary to what was observed in the East China Sea. In order to assess the sensitivity of our results to this normalization, we re-analyzed our observations using the methods of [10] and found the proportion of turtles in proximity to the peripheries of eddies was greater than eddy centers (not shown). Thus, the results are highly sensitive to whether or not the relative proportions of turtle populations in the various radial bands are normalized by area.

The tagging of adult Leatherback turtles (*Dermochelys coriacea*) has revealed that individuals spend considerable time in the interiors of anticyclonic eddies. In the Bay of Biscay in the Northeastern Atlantic, a single leatherback turtle was observed to circle the center of a large anticyclonic eddy for 33 days [50]. In the Indian Ocean, two leatherback turtles were observed to migrate southward from their nesting beaches along the coast of southeastern Africa and spend multiple days in a series of anticyclonic eddies [51, 52]. In both of these studies the authors suggested that the anticyclonic eddies were likely regions of elevated foraging success. Furthermore, the analysis in [37] suggests that anticyclones along the coast of southeastern Africa have low CHL which is consistent with the results presented here for the BMC south of 35°S.

## Summary and conclusions

By collocating the trajectories of juvenile loggerhead sea turtles with the tracks of mesoscale eddies, we provided evidence of the preferential use of the interiors of anticyclonic eddies by juvenile loggerhead turtles in the offshore region of the BMC south of 35°S. These eddies were associated with low CHL and warm SST anomalies. We present two possible mechanisms for the observed affinity of turtles towards anticyclones: (1) turtles were passively advected south of 35°S in the interiors of anticyclones, and (2) turtles were actively seeking out water masses trapped within anticyclones south of 35°S, possibly because of suitable foraging conditions, decreased predation, elevated SST, or other reasons not discernable from the data analyzed here. Inferred swimming speeds were lower in the cores and inner peripheries of anticyclones, and higher in their outer peripheries, which is consistent with a behavioral preference for the interiors of these features. Swimming speeds were also elevated in the cores of cyclones, which is suggestive of their avoidance of such features. Further targeted field studies assessing the interaction of the turtles with their prey are needed to identify the primary mechanisms resulting in the observed preference for the cores of anticyclonic eddies.

This study revealed that by combining contemporaneous satellite observations of CHL, SST, and SLA, with the trajectories of turtles, drifters and eddies, new understanding can be obtained about association of turtles with mesoscale eddies in the open ocean. This methodology can be applied to the investigation of the use of mesoscale eddies by any organism that can be tracked in space and time with precision sufficient to resolve motions at the oceanic mesoscale. This knowledge can aid in the identification of critical habitat in the open-ocean, which could be of use in the protection of endangered or over-harvested species, for example via the creation of mobile marine protected areas [53].

## Supporting information

S1 File Turtle location data. The turtle location data used in this study. Data are stored in NetCDF format.
(NC) Click here for additional data file.
